# Bedside medication review with cognitive and depression screening by a clinical pharmacist as part of a comprehensive geriatric assessment in hospitalized older patients with polypharmacy: A pilot study

**DOI:** 10.1371/journal.pone.0276402

**Published:** 2022-10-21

**Authors:** Veerle Mertens, Leen Jacobs, Nicole Knops, Seyedeh Malihe Alemzadeh, Kay Vandeven, Jo Swartenbroekx, Greta Moorkens, Maurits Vandewoude

**Affiliations:** 1 Department Geriatrics, Antwerp University Hospital, Antwerp, Belgium; 2 Faculty Pharmacy, University of Antwerp, Antwerp, Belgium; 3 Faculty Political and Social Sciences, Department Management, Belgium and University of Antwerp, Antwerp University Hospital, Antwerp, Belgium; 4 Department Pharmacy, General Hospital Sint Jozef Malle, Malle, Belgium; 5 Department Pharmacy, Antwerp University Hospital, Antwerp, Belgium; 6 Department Internal Medicine, Antwerp University Hospital, Antwerp, Belgium; Jouf University, UNITED KINGDOM

## Abstract

**Background:**

Polypharmacy is highly prevalent in older patients with multimorbidity and is associated with increased risk of adverse drug events. This pilot study investigated the added value of a bedside medication review with cognitive and depression screening by a clinical pharmacist to identify potentially inappropriate medications (PIMs) and medication use issues in older patients with polypharmacy.

**Methods and results:**

In the period from September 2018 to March 2019, a clinical pharmacist took part in the comprehensive geriatric assessment of 37 older patients hospitalized at Antwerp University Hospital and conducted a medication review consisting of a record review, a bedside interview questionnaire covering medication use, evaluation of cognitive function (abbreviated MMSE), depression (GDS-4), and systematic check for possible PIMs (STOPP/START criteria).

Patients were 83±4 years old and on a median of 12 home medications (range 5–20). The clinical pharmacist formulated an average of 7.7 recommendations to optimize medication use per patient, of which 89.9% were considered clinically relevant by the geriatrician. Only 2 out of 286 PIMs were discovered during routine electronic validation of medication prescriptions. Supervision of medication intake was absent in 75% of cognitively impaired patients, but advice to do so was implemented in 86.4% of cases. The multidisciplinary geriatric advice was communicated to the treating physician, who fully implemented 33.8% of the recommendations.

**Conclusions:**

Bedside medication review with cognitive and depression screening by a clinical pharmacist is useful to discover polypharmacy related problems and medication intake issues in a population of geriatric patients. Systematic screening for cognitive impairment and depression are needed to detect patients in need of support for correct medication use and therapy compliance.

## Introduction

An aging population brings with it a higher prevalence of multimorbidity and the need for more pharmacological therapy. Polypharmacy, defined as the chronic use of 5 or more different drugs [1], becomes increasingly common among older persons. Ample evidence exists that polypharmacy is associated with increased medication safety issues and an increased risk of adverse drug events (ADEs).

Polypharmacy has been shown to increase the risk of drug interactions, drug toxicity, falls, delirium and nonadherence [2], as well as increasing hospitalization rate and mortality risk [3]. This is especially the case in older patients, as changes in the pharmacokinetics and pharmacodynamics make older people more vulnerable for drug-related harm [4].

A number of validated assessment tools can be used to optimize medication use and provide guidance in determining medication appropriateness in the elderly [5]. These tools can be categorized into explicit, criterion based tools such as the Beers criteria [6] and the Screening Tool of Older Person’s Prescriptions (STOPP) / Screening Tool to Alert doctors to Right Treatment (START) criteria [7]; implicit, judgment based tools such as the Medication appropriateness Index (MAI) [8] and tools combining these approaches. [5] A 2018 systematic review found that these tools resulted in a reduction of inappropriate prescribing, but there was conflicting evidence regarding the effect of a medication review using these tools on clinical outcomes [9]. However, individual studies did find a positive effect of medication reviews on clinical parameters such as emergency department visits [10], hospital readmissions [10,11] or quality of life [12]. Successful deprescribing interventions have been reported for interventions by physicians, clinical pharmacists and multidisciplinary teams [13].

Here we report the results of a pilot study investigating the value of bedside medication review by a clinical pharmacist as part of a multidisciplinary geriatric team to detect potentially inappropriate medication (PIM) and issues with medication use in older patients with polypharmacy, hospitalized on non-geriatric wards of a tertiary hospital. The main objectives of the study were to determine whether a patient interview with cognitive and depression screening by a clinical pharmacist has an added value over routine medication prescription checks to reveal PIMs and medication use issues, and whether the recommendations by the clinical pharmacist are clinically relevant in a geriatric patient population.

## Methods

Geriatric patients hospitalized in the cardiology or orthopedics departments of Antwerp University Hospital in the period from September 2018 to March 2019 were selected on predefined days on which the clinical pharmacist was available. Antwerp University Hospital is a tertiary hospital without a dedicated geriatric ward. Its core team for multidisciplinary geriatric consultations normally consisting of a geriatrician and a nurse, was expanded with a clinical pharmacist for this study.

The inclusion criteria were age ≥ 75 years (cut-off for reimbursement of geriatric assessment in Belgian social security system) and polypharmacy, i.e., home medication consisting of ≥5 drugs, excluding skin care products and certain over the counter drugs such as artificial tears or homeopathy. Only patients expected to remain admitted in the hospital for at least two more days after the pharmaceutical consultation were eligible for inclusion in the study. Exclusion criteria were communication issues due to a language barrier or serious confused state of the patient. The study was approved by the Ethical Committee of Antwerp University Hospital and patients gave written informed consent before inclusion.

For every included patient, a pre-consultation record review, pharmaceutical bedside interview, and a comprehensive medication review were performed by the clinical pharmacist.

### Patient record review

The medical record of included patients was reviewed with specific attention to the reason for hospitalization, medical history of the patient, allergies, home medication and medication taken during hospitalization. Relevant laboratory values such as kidney function parameters, INR and potassium level were noted. Data retrieved during the record review were available to the clinical pharmacist during the interview with the patient.

### Pharmaceutical bedside interview

A standardized questionnaire, developed collaboratively by the clinical pharmacist and the geriatrician, was used to interview the patient on general medication use topics, their knowledge of the medications they were taking, and practical issues related to their use.

To assess the patients’ knowledge of their medication, they were asked to name the indication or reason for prescription for two of the drugs of choice on their medication list.

Additionally, cognitive functioning was assessed via an abbreviated version of the mini-mental state examination (MMSE) [14], and patients were screened for depression using the geriatric depression scale 4 (GDS-4) [15]. The abbreviated MMSE covered the short-term (3items) and long-term memory (3items) components and the attention component (5 items) of the MMSE [14]. A score of 2/3 or lower on the long-term memory component of the abbreviated MMSE was considered as cognitive decline, patients with a score higher than 1 on the GDS-4 were referred for further follow-up of depressive symptoms. The clinical pharmacist had been trained in performing the MSSE and GDS-4 screenings.

### Medication review

The STOPP/ START- criteria [7] were used to perform an in-depth medication review checking for each medication whether the prescribed drug was the right choice for a particular indication, taking into account the multi-morbidity of the geriatric patient.

A recommendation was formulated for every potentially inappropriate medicine (PIM) discovered. These recommendations were classified into 12 different categories:

1. Dose: recommendations on dose adjustments.
2. No indication: recommendations on discontinuation of treatment or re-evaluation due to the lack of an indication.
3. Stop: recommendations on discontinuation of treatment for reasons other than increased risk of falling (according to the STOPP- criteria).
4. Start: recommendations on the initiation of drugs that are considered necessary but have not yet been administered (according to the START- criteria).
5. Stop due to increased risk of falling: recommendations on discontinuation of treatment due to increased risk of falling.
6. Interaction: recommendations on drug interactions.
7. Contra-indication: recommendations on contra-indications. Possible alternatives were recommended.
8. Time of intake: recommendations on the time of intake of specific drug(s).
9. Discrepancy: recommendations on discovered discrepancies between home medication and hospital-initiated medication.
10. Switch: change of formulation was recommended.
11. Monitoring: therapeutic drug monitoring was recommended. Measurements at defined intervals to monitor renal function, blood ion values and/ or drug concentrations. (For example: monitoring of a patient’s potassium level during the start-up of two potassium-sparing medicines).
12. Changes related to practical use / depression / cognition: groups various recommendations related to practical issues with medication use or to patient cognition or depression. (For example: providing a pill splitter or a magistral preparation to facilitate intake or suggest home nursing assistance or supervision of medication preparation and intake in case of cognitive decline).

Each recommendation was briefly motivated and registered as an intervention. Multiple recommendations for one drug were registered as multiple interventions in the registration file.

### Validation of recommendations by the geriatrician

To assess the clinical relevance of the recommendations made by the clinical pharmacist, the geriatrician reviewed the patient chart, and interviewed and performed a clinical evaluation of the patient. The geriatrician then reviewed and either accepted or rejected the recommendations formulated by the clinical pharmacist, based on their potential clinical impact using the tool of Chedru and Juste [16]. Only recommendations not judged to be clinically relevant for the current patient were rejected. A short motivation was given in the registration file for rejected recommendations.

### Reporting and implementation follow-up

Recommendations accepted by the geriatrician were reported to the treating physician in the hospital. Patient records were reviewed to check the implementation of the recommendations by the treating physician two days after the recommendation or after patient discharge.

### Data collection and analysis

Patient data collected included name and patient number, hospital ward, medical discipline, anamnesis, reason of admission, relevant laboratory parameters, home medication, medication started or paused in the hospital, questionnaire responses. The time spent conducting the bedside interview and the medication review was also documented. The ADEs detected during the bedside medication review process followed in this study were compared to the ADEs detected during the routine medication validation that is performed in the electronic prescribing system as part of the standard of care at the hospital. Data are expressed as percentages, mean ± standard deviation or median (range). The data were processed using Microsoft Office Excel 2016.

The association of START/STOPP recommendations or PIMs for certain drug classes with screening results suggestive for depression or cognitive impairment, and with supervision on the medication intake were analyzed with Pearson’s chi square test using SPSS statistics software. Significance was accepted for p<0.05.

## Results

### Population characteristics

This pilot study included 37 patients, 11 hospitalized in the orthopedics department and 26 in the cardiology department of Antwerp University Hospital, a tertiary care facility without a dedicated geriatric ward. Included patients were on average 83±4 years old and were using on average 11.6 different home medications (median 12, range 5–20).

A decreased cognitive status, as judged by a less than perfect score on the long-term memory component of the abbreviated MMSE, was observed in 24 of 37 patients (64.9%). Remarkably, 18 of 24 cognitively impaired patients (75%) were responsible for preparing their home medication themselves. Lower short-term memory scores were found in 2 patients (5.4%), whereas diminished attention was observed in 11 patients (29.8%).

Based on the depression screening, four of 37 patients (10.8%) were referred to further follow-up for depression. Supervision on medication intake in patients referred for depression follow-up was already organized for two patients and was recommended and implemented after the medication review for the other two patients.

### Medication use

During the bedside interview, patients were questioned on general aspects of medication use, as well as practical issues concerning medication use. Thirty-five out of 37 patients (94.6%) reported having a regular home pharmacist.

Assistance in procuring medication was reported by 14 patients (38.9%), while 12 patients (33.3%) reported receiving assistance in preparing their medication, mostly by family members (n = 5, 13.9%) or a home nurse (n = 2, 5.4%).

Seven patients (18.9%) reported taking their medication under supervision. Supervision of medication preparation or use was recommended for 13 patients (29.7%) as a result of the bedside interview by the clinical pharmacist. This recommendation was implemented by the treating physician for 11 out of 13 (84.6%) patients. In four patients, a recommendation for supervision of medication intake through home nursing was documented in the patient record but was not reported by the patient during the bedside interview. Supervision of medication intake was associated with significantly less medications for which no current indication existed (p = 0.028) and with significantly less PIMs for gastro-intestinal medications (p = 0.016).

Thirty-five patients (94.6%) reported always taking their medications as prescribed by the physician. Twenty-three patients (62.2%) reported having a medication scheme. Twenty-eight patients (75.7%) could name the indication(s) for two drugs of their own choosing on their medication list.

The bedside interview with the patient revealed that 9 out of 37 patients (24.3%) had practical issues with medication use, with problems pushing small tablets out of the blister and difficulty reading the small print on the outer or inner packaging as the most frequently reported problems.

### Medication review data

**Fig 1:**
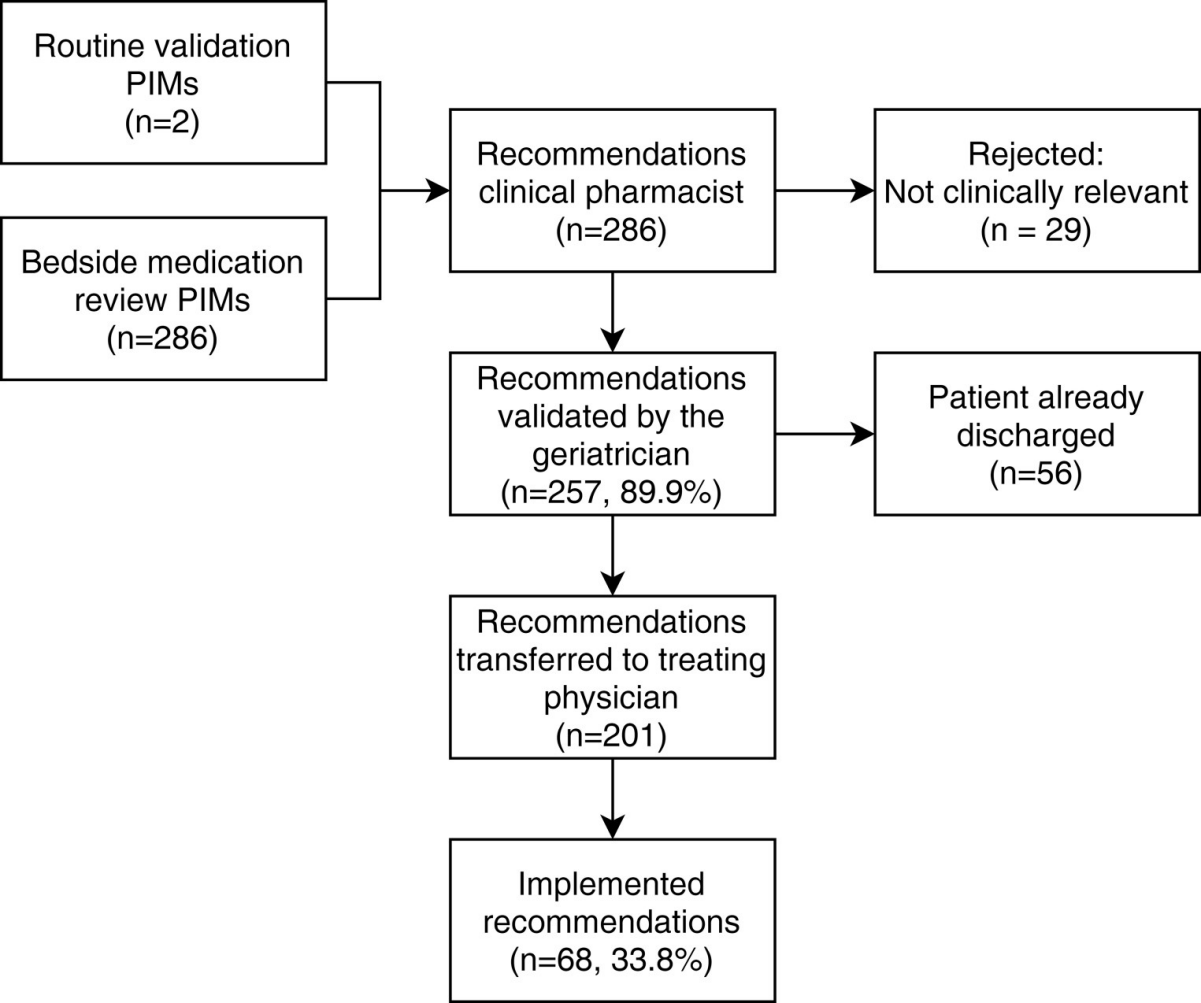
Flowchart of recommendations by the pharmacist: 89.9% of recommendations were validated as clinically relevant by the geriatrician. Of the 201 recommendations passed on to the treating physician, only 68 (33.8%) were implemented. *PIM*: *Potentially inappropriate medicine*.

**Fig 1** is a flowchart of the results of the medication review. A total of 286 PIM-related recommendations were formulated, with an average of 7.7 (SD: 3.1, range 5–17) per patient; for just 2 patients, no medication optimization recommendations were given. Only 2 of the total number of PIMS identified by the clinical pharmacist after the bedside medication review were discovered during routine electronic validation of the medication prescriptions.

**Fig 2:**
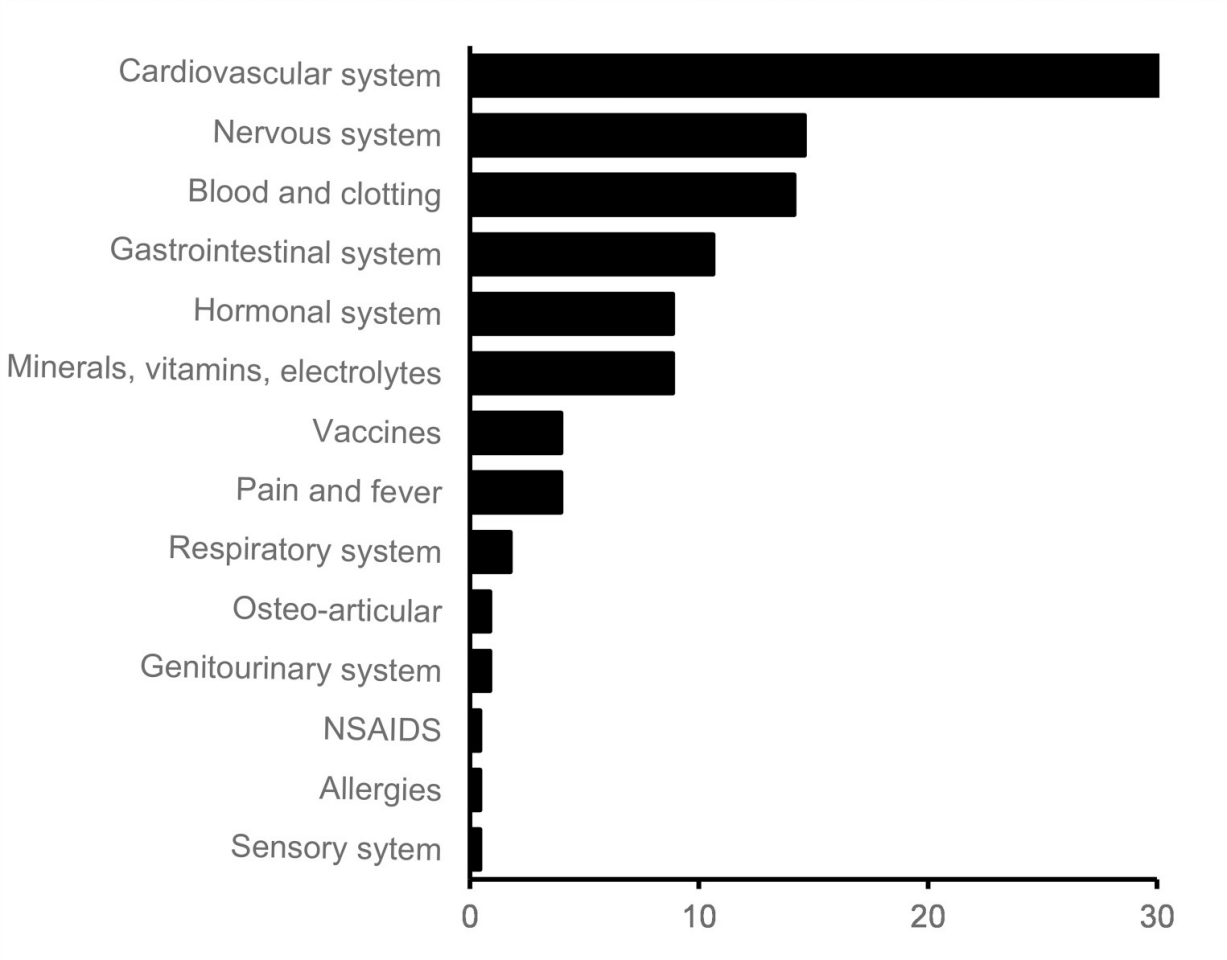
PIMs by drug class. Recommendations made by the clinical pharmacist after the bedside medication review, summarized by drug class. Data are expressed as %. *PIM*: *Potentially inappropriate medicine*.

The identified PIMS mainly concerned medication for the cardiovascular system (30.09%), nervous system (14.6%) and drugs affecting the blood and clotting system (14.2%). The distribution of the discovered PIM’s over different drug classes is depicted in Fig 2.

In patients with a GDS-4 score suggestive of depression, less PIMs for pain and fever medication were found (p = 0.036), whereas in patients who took their medication under supervision, significantly less PIMS related to gastro-intestinal drugs were detected (p = 0.016).

**Fig 3:**
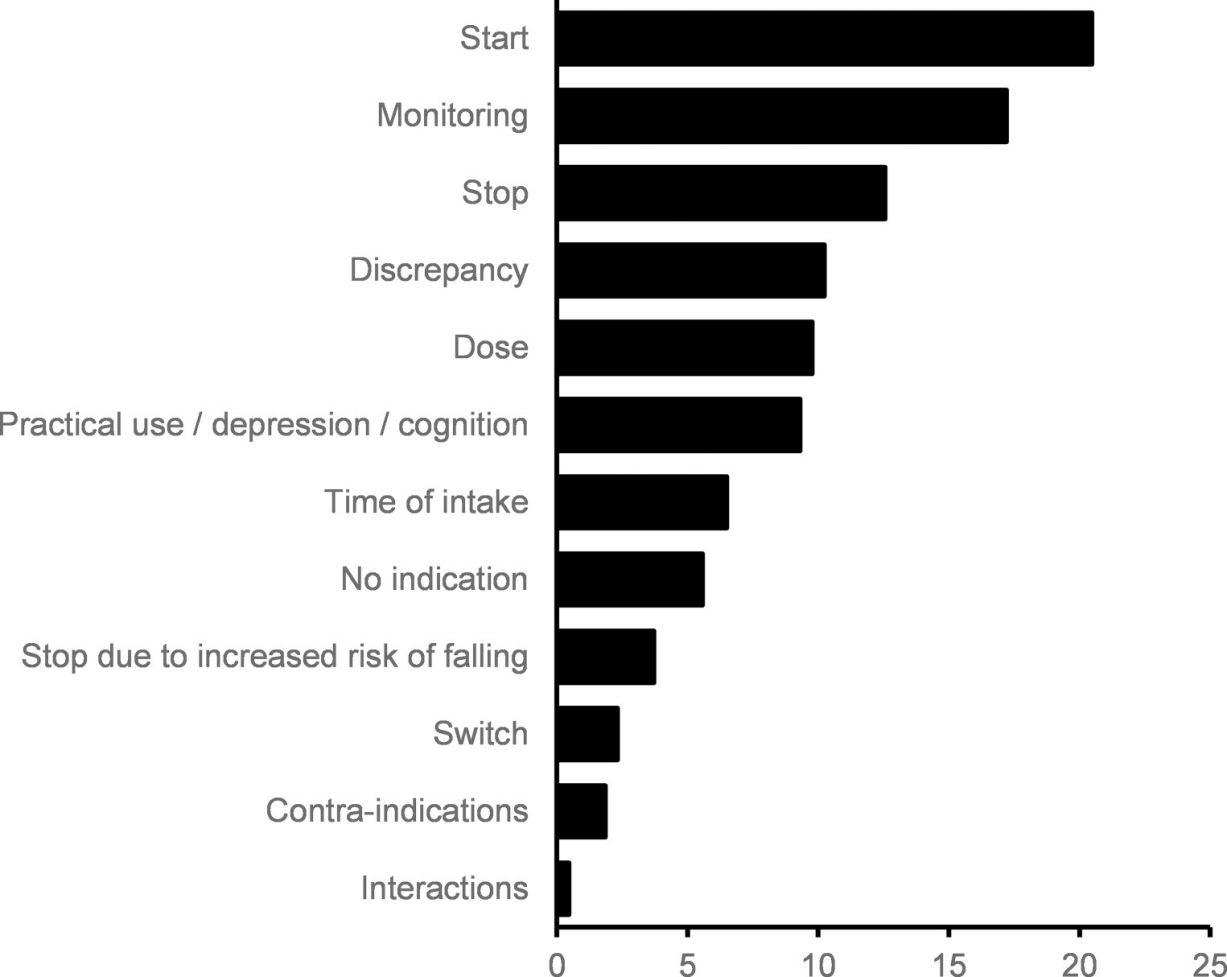
Recommendation types. Recommendations made by the clinical pharmacist after the bedside medication review, summarized by type of recommendation. Data are expressed as %.

Recommendations made by the clinical pharmacist were grouped into 12 categories, as shown in Fig 3. Most of them related to necessary and missing medications (start recommendation: 20.5%), to the need for monitoring (17.2%) or the need to stop a certain medication (12.6%).

Screening results indicative of cognitive impairment were associated with a significantly higher number of discrepancies between home and hospital-initiated medications (p = 0.031) and more PIMs related to interactions (p = 0.031).

The pharmaceutical bedside interview lasted an average of 47±16 minutes and the medication review lasted an average of 68±16 minutes, not taking into account waiting times for the clinical pharmacist, e.g. due to sleeping patients, another examination or treatment in process, absence of the patient on the ward.

### Validation by the geriatrician

After examining the patient, the geriatrician reviewed the recommendations of the clinical pharmacist. Of the 286 recommendations formulated by the clinical pharmacist, 257 (89.9%) were accepted and considered clinically relevant by the geriatrician; the remaining recommendations were not considered as having a clinical impact.

The geriatric consultation could not take place in 11 patients due to early discharge of the patient, resulting in 56 recommendations that could not be transmitted to the treating physician during the patient’s hospitalization. For these patients, the geriatrician informed the general practitioner by phone about the medication recommendations.

### Implementation

Only 68 out of 201 recommendations (33.8%) made after the bedside medication review process were accepted and implemented by the treating physician in the hospital (Fig 1).

## Discussion

In this pilot study, a comprehensive medication review was performed by a clinical pharmacist in older patients with polypharmacy hospitalized on the cardiology or orthopedics wards of a tertiary hospital, using the STOPP/START criteria to evaluate the appropriateness of prescribed medications. The clinical pharmacist interviewed the patient to detect practical issues with medication use and screen for both cognitive impairment and depressive symptoms.

The medication review resulted in on average 7.7 recommendations per patient for optimizing medication use, whereas only two of the total number of recommendations were also discovered during routine validation using the hospital’s electronic prescribing system. Routine prescription validation mainly checks the correctness of the dose, time and route of administration, interactions, contra-indications, allergic reactions, and treatment duration, but does not take into account indications or clinical parameters The suitability of the medication is therefore not thoroughly validated. For example, the daily routine validation does not consider omission of essential medications, monitoring of blood values and therapeutic drug monitoring (except for vancomycin levels). Neither are cognitive problems or practical difficulties in the use of the medication considered during this routine validation step. The majority of patients reported having a home pharmacist, but under the Belgian social security system, the role of the home pharmacist, established in October 2017, is that of a community pharmacist with only the additional responsibility of keeping the patient’s medication scheme up to date. Official registration of the home pharmacist in the social security system was not double checked for the patients included in this study.

The two most formulated recommendations in this study involved the initiation of a necessary treatment and the need for monitoring of blood levels or body parameters. The drug classes in which most PIMs were discovered also differed strongly between routine validation and extensive medication review. Routine validation mainly focuses on PIMs in the class of antimicrobials, blood/coagulation, and pain /fever medications. While in the comprehensive medication review, PIMs were mainly discovered in the cardiovascular system, nervous system, and blood/ coagulation drug classes. An explanation for this may be that most patients included in this study were admitted to the cardiology department.

Despite its small population size, the relatively large number of recommendations per patient (range 5–17) in this study, with recommendations made for 35 out of 37 patients, indicates that systematically reviewing the medication in older polypharmacy patients is important to optimize medication use and prevent ADEs. Multidisciplinary geriatric teams in Belgium in general include a geriatrician, a nurse and an occupational therapist, but no clinical pharmacist.

The short cognitive and depression screening by the clinical pharmacist proved a valuable part of the bedside interview to be able to start supervision on medication intake, either by professionals such as a home nurse or by family caregivers in patients with cognitive impairment or (suspected) depression. When alerted to cognitive impairment or depressive symptoms in their patients, treating physicians in the hospital implemented the recommendation to organize supervision on medication intake after discharge from the hospital in most cases. Noteworthy, the limited screening performed in this study identified patients with signs of depression and cognitive impairment that had remained unnoticed. Signs of depression or cognitive impairment, as well as the need for supervision of medication intake, had a significant influence on the type of PIMs detected in the medication review, with less PIMs for pain medication in patients screening positive for depression, whereas in cognitively impaired patients, a discrepancy between home and hospital medication and interaction PIMs were detected more often. Supervision of medication intake was associated with less no indication PIMs and less PIMs related to gastro-intestinal medication.

Since patients in a delusional state were explicitly excluded from the study, temporary confusion or delirium are not likely to be responsible for the relatively high proportion of cognitive impairment we observed.

Our findings suggest that systematic screening for depression and cognitive impairment would be a useful addition to the standard care of hospitalized older patients for all types of hospitalizations. This type of limited screening, performed in this study by the clinical pharmacist, requires only limited resources and could be delegated to several functions within the care team. It is important to raise awareness of physicians and caretakers they need to check whether patients are capable of taking their medication correctly and organize support if this is not the case.

While the geriatrician accepted 89.9% of the medication change recommendations of the clinical pharmacist as clinically relevant, only 33.8% of these recommendations were implemented by the treating physicians. A similarly low implementation rate (42%) was observed in a retrospective study evaluating 100 randomly selected medication reviews in the acute medical ward of a Danish Hospital. Implementation rates of medication review recommendations were found to be higher when communicated earlier and verbally to the treating physician. The type of recommendations implemented most frequently in that study were recommendations to discontinue drugs for which there was no longer an indication, for inappropriate drug formulations or drug doses being too low [17]. In a 2009 Belgian study, the reported clinical relevance of the recommendations made by the pharmacist was comparable to that in our study (87.8%), but the implementation rate was much higher (84% of treatment changes persisted 3 months after discharge), possibly because that study was conducted in patients hospitalized in a geriatric ward [18], in contrast to our study which included patients from the orthopedic and cardiology wards. A similarly high acceptance rate of clinical pharmacists’ intervention was reported in a Turkish study of geriatric ward inpatients in a teaching hospital [19].

Possible explanations for the low implementation rate of the recommendations made in our study include limited experience and expertise in geriatrics of the orthopedic and cardiology ward physicians, as monitoring of patients in a university hospital is mostly done by doctors in training under supervision of a specialist, lack of time, or quick discharge of the patient leaving insufficient time to make changes to the home medication. A 2014 review of qualitative research lists knowledge gaps, lack of evidence, insufficient time, respecting other prescribers’ autonomy and fearing negative consequences of changing existing medications as barriers for physicians to minimize PIMs [20]. In an ongoing follow-up study, we investigate whether participation of the clinical pharmacist in a weekly clinic round can increase the implementation rate.

The bedside interview further revealed that 24.3% of patients experienced practical problems using their medication. Such problems can negatively affect treatment adherence and increase the risk of ADEs. Skipping medication intake can potentially deteriorate the medical condition of patients, potentially resulting in a prescription cascade that further increases polypharmacy and the associated ADE risk. Discovering these practical problems was only made possible through the direct contact between clinical pharmacist and patient.

The most important limiting factor for the implementation of a bedside medication review in daily practice is the time constraint. A medication review took an average of 68 minutes and the pharmaceutical consultation took an average of 47 minutes, while it was initially expected that the consultation would take about 20 minutes [21], although a similar total time investment of 2 hours per patient has also been reported [22].

Often, the lonely and very emotional older people saw the bedside interview as an opportunity to start a wide-ranging conversation. The personal questions of the depression screening were often the catalyst for this. The clinical pharmacist’s inexperience with anamnesis and direct contact with patients, compounded with a lack of patient communication training in their training curriculum, also contributed to the time needed for the interviews. This is in line with the findings of van Eikenhorst et al. [23] who made video observations of clinical pharmacists during patient interviews and found that pharmacists mostly responded non-explicitly to negative emotions voiced by the patient. They recommend that clinical pharmacists must be trained in patient communication to give more explicit responses to the patient and acquire more in-depth insight into the patient’s problems [23].

## Conclusions

This pilot study demonstrated that medication review by a clinical pharmacist can contribute to optimal medication use in older patients with polypharmacy, especially in combination with screening for cognitive functioning and depression. Bedside interviews by the clinical pharmacist have the added value of revealing practical problems with medication use that may contribute to nonadherence or increased risk of ADEs. This approach entails a considerable time investment of the team and needs to be optimized further to increase the implementation by the treating physician of the prescribing recommendations made. Important calls to action for the future are additional education of physicians on polypharmacy and medication review, increased awareness regarding the importance of systematically screening patients for cognitive dysfunction and depression, and patient communication training for clinical pharmacists.

## References

[1] Masnoon N, Shakib S, Kalisch-Ellett L, Caughey GE. What is polypharmacy? A systematic review of definitions. BMC Geriatr. BMC Geriatrics; 2017;17:1–10. https://doi.org.10.1186/s12877-017-0621-2. 29017448 10.1186/s12877-017-0621-2 PMC5635569

[2] Hoel RW, Giddings Connolly RM, Takahashi PY. Polypharmacy Management in Older Patients. Mayo Clin Proc [Internet]. Mayo Foundation for Medical Education and Research; 2021;96:242–56. https://doi.org.10.1016/j.mayocp.2020.06.012. 10.1016/j.mayocp.2020.06.012 33413822

[3] Hilmer S, Gnjidic D. The Effects of Polypharmacy in Older Adults. Clin Pharmacol Ther [Internet]. 2009;85:86–8. doi: 10.1038/clpt.2008.224 19037203

[4] Lavan AH, Gallagher PF, O’Mahony D. Methods to reduce prescribing errors in elderly patients with multimorbidity. Clin Interv Aging. 2016;11:857–66. doi: 10.2147/CIA.S80280 27382268 PMC4922820

[5] Kaufmann CP, Tremp R, Hersberger KE, Lampert ML. Inappropriate prescribing: A systematic overview of published assessment tools. Eur J Clin Pharmacol. 2014;70:1–11. doi: 10.1007/s00228-013-1575-8 24019054

[6] Fick DM, Semla TP, Steinman M, Beizer J, Brandt N, Dombrowski R, et al. American Geriatrics Society 2019 Updated AGS Beers Criteria® for Potentially Inappropriate Medication Use in Older Adults. J Am Geriatr Soc. 2019;67:674–94. doi: 10.1111/jgs.15767 30693946

[7] O’mahony D O’sullivan D, Byrne S, O’connor MN, Ryan C, Gallagher P. STOPP/START criteria for potentially inappropriate prescribing in older people: Version 2. Age Ageing. 2015;44:213–8. doi: 10.1093/ageing/afu145 25324330 PMC4339726

[8] Hanlon J, Schmader K, Samsa G, Weinberger M, Uttech K, Lewis I. A method for assess drug therapy appropriateness. J Clin Epidemiol. J CUB Epiaemiol. 1992;45:1045–1051. 10.1016/0895-4356(92)90144-c 1474400

[9] Cooper JA, Cadogan CA, Patterson SM, Kerse N, Bradley MC, Ryan C, et al. Interventions to improve the appropriate use of polypharmacy in older people: A Cochrane systematic review. BMJ Open. 2015;5. doi: 10.1136/bmjopen-2015-009235 26656020 PMC4679890

[10] Renaudin P, Boyer L, Esteve MA, Bertault-Peres P, Auquier P, Honore S. Do pharmacist-led medication reviews in hospitals help reduce hospital readmissions? A systematic review and meta-analysis. Br J Clin Pharmacol. 2016;82:1660–73. doi: 10.1111/bcp.13085 27511835 PMC5099542

[11] Gillespie U, Alassaad A, Hammarlund-Udenaes M, Mörlin C, Henrohn D, Bertilsson M, et al. Effects of Pharmacists’ Interventions on Appropriateness of Prescribing and Evaluation of the Instruments’ (MAI, STOPP and STARTs’) Ability to Predict Hospitalization-Analyses from a Randomized Controlled Trial. PLoS One. 2013;8. doi: 10.1371/journal.pone.0062401 23690938 PMC3656885

[12] Van der Linden L, Decoutere L, Walgraeve K, Milisen K, Flamaing J, Spriet I, et al. Combined Use of the Rationalization of Home Medication by an Adjusted STOPP in Older Patients (RASP) List and a Pharmacist-Led Medication Review in Very Old Inpatients: Impact on Quality of Prescribing and Clinical Outcome. Drugs and Aging. Springer International Publishing; 2017;34:123–33. doi: 10.1007/s40266-016-0424-8 27915457

[13] Gnjidic D, Le Couteur DG, Kouladjian L, Hilmer SN. Deprescribing trials: methods to reduce polypharmacy and the impact on prescribing and clinical outcomes. Clin Geriatr Med [Internet]. Elsevier; 2012;28:237–53. https://doi.org.10.1016/j.cger.2012.01.006. 10.1016/j.cger.2012.01.006 22500541

[14] Folstein MF, Folstein SE, McHugh PR. “Mini-mental state”. A practical method for grading the cognitive state of patients for the clinician. J Psychiatr Res. 1975;12:189–98. doi: 10.1016/0022-3956(75)90026-6 1202204

[15] D’Ath P, Katona P, Mullan E, Evans S, Katona C. Screening, detection and management of depression in elderly primary care attenders—I: The acceptability and performance of the 15 item Geriatric Depression Scale (GDS15) and the development of short versions. Fam Pract. 1994;11:260–6. https://doi.org.10.1093/fampra/11.3.260. 7843514 10.1093/fampra/11.3.260

[16] Chedru V, Juste M. Evaluation médicale de l’impact clinique des interventions pharmaceutiques. J Pharm Clin. 1997;16:254–8.

[17] Bülow C, Færch KU, Armandi H, Jensen BN, Sonne J, Christensen HR, et al. Important Aspects of Pharmacist-led Medication Reviews in an Acute Medical Ward. Basic Clin Pharmacol Toxicol. 2018;122:253–61. doi: 10.1111/bcpt.12901 28871627

[18] Spinewine A, Dhillon S, Mallet L, Tulkens PM, Wilmotte L, Swine C. Implementation of ward-based clinical pharmacy services in Belgium-description of the impact on a geriatric unit. Ann Pharmacother. 2006;40:720–8. doi: 10.1345/aph.1G515 16569792

[19] Ertuna E, Arun MZ, Ay S, Koçak FÖK, Gökdemir B, İspirli G. Evaluation of pharmacist interventions and commonly used medications in the geriatric ward of a teaching hospital in turkey: A retrospective study. Clin Interv Aging. 2019;14:587–600. doi: 10.2147/CIA.S201039 30962679 PMC6432892

[20] Anderson K, Stowasser D, Freeman C, Scott I. Prescriber barriers and enablers to minimising potentially inappropriate medications in adults: A systematic review and thematic synthesis. BMJ Open. 2014;4. doi: 10.1136/bmjopen-2014-006544 25488097 PMC4265124

[21] Choukroun C, Leguelinel-Blache G, Roux-Marson C, Jamet C, Martin-Allier A, Kinowski JM, et al. Impact of a pharmacist and geriatrician medication review on drug-related problems in older outpatients with cancer. J Geriatr Oncol [Internet]. Elsevier Inc.; 2021;12:57–63. doi: 10.1016/j.jgo.2020.07.010 32800700

[22] Gutiérrez-Valencia M, Izquierdo M, Beobide-Telleria I, Ferro-Uriguen A, Alonso-Renedo J, Casas-Herrero Á, et al. Medicine optimization strategy in an acute geriatric unit: The pharmacist in the geriatric team. Geriatr Gerontol Int. 2019;19:530–6. doi: 10.1111/ggi.13659 30950148

[23] van Eikenhorst L, van Dijk L, Cords J, Vervloet M, de Gier H, Taxis K. Pharmacists’ responses to cues and concerns of polypharmacy patients during clinical medication reviews—A video observation study. Patient Educ Couns [Internet]. Elsevier Ireland Ltd; 2020;103:930–6. https://doi.org.10.1016/j.pec.2019.11.032. 10.1016/j.pec.2019.11.032 31859122

